# Sacral agenesis without maternal diabetes: a case report

**DOI:** 10.1097/MS9.0000000000001277

**Published:** 2023-09-05

**Authors:** Jagadish Thapa, Abhishek Pandey, Archana Pandey, Suraj Keshari, Karuna Bista, Aashutosh Chaudhary

**Affiliations:** a Kathmandu University School of Medical Sciences, Dhulikhel Hospital, Dhulikhel; b Nepal Medical College, Attarkhel, Gokarneshwor, Nepal

**Keywords:** case report, caudal regression syndrome, congenital abnormalities, sacral agenesis

## Abstract

**Introduction:**

Sacral agenesis is a rare congenital condition that is characterized by sacrococcygeal bone agenesis. It is associated with spinal cord anomalies as well as problems with the genitourinary system, large bowel, and lower extremities. Fetal ultrasound allows for diagnosis even before birth.

**Case presentation:**

The authors present the case of a 1-year-old girl with sacral agenesis type III and bilateral congenital talipes equinovarus with spina bifida who was born to a nondiabetic mother and had a normal anomaly scan.

**Clinical discussion:**

People with less severe forms of sacral agenesis can live a normal life, and it is not connected with cognitive impairment; however, concomitant bladder, colon, and lower limb disorders cause considerable morbidity. The majority of treatment is supportive, frequently requiring orthopedic, urological, gastroenterological, pediatric, and physiotherapy support.

**Conclusion:**

Genetic and prepregnancy counseling, as well as early screening of high-risk mothers, remain the only options for prevention of the disease since treatment is mostly supportive.

## Introduction

Highlights

- Sacral agenesis can occur in children with nondiabetic mothers.
- As diagnosis in the first trimester is difficult due to poor sacral ossification, the diagnosis is usually made in the later stages of pregnancy. As a result, follow-up scans are required.
- Despite being an uncommon disorder, sacral agenesis is linked to serious spinal, orthopedic, gastrointestinal, and urological problems that can cause severe morbidity.

Sacral agenesis syndrome, also known as caudal regression syndrome (CRS) or caudal dysplasia, is an uncommon congenital abnormality with an incidence of 0.1 to 0.25 per 10 000 pregnancies^1^. Maternal diabetes increases the risk of developing sacral agenesis, with reports of a 200-fold rise in incidence^2,3^. This condition develops when a portion of the spine and spinal cord fails to grow and is commonly accompanied by musculoskeletal, gastrointestinal, and genitourinary issues^4^.

We provide a case of a baby with sacral agenesis born to a nondiabetic mother who presented to our tertiary care center. The case is being reported for its rarity and educational value. This case report has been reported in line with the Surgical CAse REport (SCARE) Criteria^5^.

### Case details

A 1-year-old female child with bilateral lower limb deformities since birth, was referred to the orthopedic OPD of our tertiary-level hospital for needful orthopedic evaluation and management. She was born of a nonconsanguineous marriage, weighed 2200 g at birth, and was delivered by cesarean section for fetal bradycardia.

After the birth of her first child, the mother describes having an induced, medical termination of pregnancy 8 years ago, a few years following which she planned to have a child but developed secondary subfertility. After frequent follow-up to the gynecologist for secondary subfertility, she had a planned pregnancy. The mother began taking folic acid before conceiving; however, she did mention vomiting throughout the first trimester. She started having antenatal visits at 6 weeks of pregnancy. She had 12–14 antenatal hospital visits throughout the pregnancy. Her random blood glucose at 20 weeks was 93 mg/dl (5.16 mmol/l). Fetal ultrasound was performed two times, and an anomaly scan was performed at five months, all of which reported normal findings. The mother had gestational hypertension diagnosed at 40 weeks of gestation. There is no exposure to any drugs like sodium valproate, retinoic acid, or minoxidil.

She is the second child, with the first child being around 9 years older with no similar illness. The mother and father were 26 and 29 years old, respectively, at conception, with a history of diabetes only in a paternal granduncle as shown in Table 1. There is no history of congenital anomalies or inheritable diseases in her family.

**Table 1:**
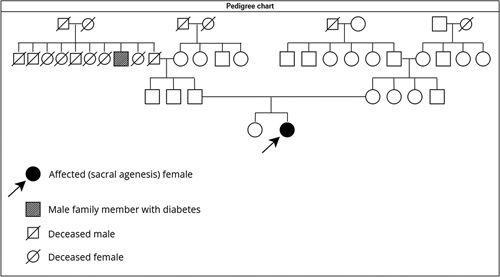
Pedigree chart showing four generations of the family.

She was diagnosed with bilateral secondary congenital talipes equinovarus (clubfoot) with a Pirani score of 5.5 and left hip subluxation secondary to sacral agenesis at birth. Repeated studies of the urinary and intestinal tracts have been reported as normal.

**Figure 1:**
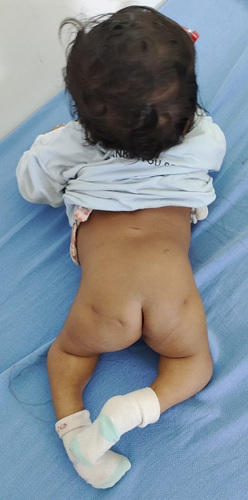
A dimple is present at the lumbosacral junction.

On examination, the patient’s weight and height were appropriate for her age. The patient was fair with the presence of bilateral clubfoot and a sacral pit at the lumbosacral junction (Fig. 1). The genitalia looked normal. Both lower limbs appeared short with flexion contractures of the knee and hip. Her sacral and coccygeal bones could not be palpated. There was decreased tone in the lower limbs, with normal tone in the upper limbs. Ankle and plantar reflexes were difficult to detect during neurological evaluation. There was decreased sensation in the bilateral lower limbs. The anal sphincter was slightly diminished in tone with fecal and urinary incontinence while crying.

### Investigations

**Figure 2:**
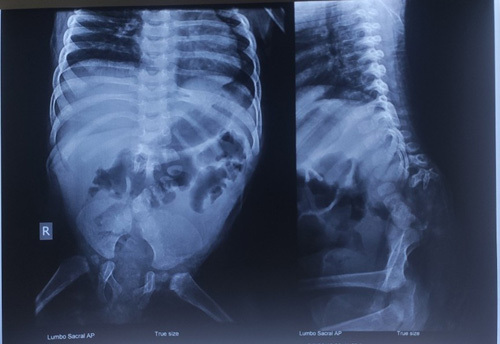
Radiograph of the spine shows incomplete fusion of the iliac bones with a left-sided shift of the lumbar vertebrae. The lowermost lumbar vertebra is articulating with the left iliac bone.

**Figure 3:**
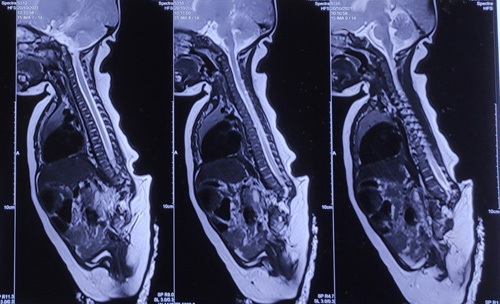
MRI spine sagittal section shows dilatation of the spinal canal of the terminal spinal cord for a distance of 2.5 cm with a maximum diameter of 2.1 mm, suggestive of syrinx. Sacrum not visualized. The iliac bones are nearly fused with each other, along with a left-sided shift of the lumbar vertebrae. The lowermost lumbar vertebra is articulating with the left iliac bone. There is evidence of a defect in the posterior arch of the L4 and L5 vertebrae without meningocele, which is suggestive of spina bifida occulta.

A skeletal survey showed complete agenesis of the sacral and coccygeal vertebrae, and hypoplastic lumbar vertebrae with incomplete fusion of the iliac bones (Fig. 2). Further investigation with an MRI spine revealed sacral agenesis type III as per Renshaw classification^6^ (Fig. 3). An ultrasound of the abdomen and pelvis ruled out any associated visceral anomalies.

### Treatment

The majority of the therapy is supportive. The child is planned for fusion surgery at the age of four, as well as congenital talus equinovarus correction and bilateral knee contracture release in the future. Meanwhile, every 4 months, urodynamic monitoring will be performed. Regular follow-up appointments with pediatricians, gastroenterologists, urologists, and physiotherapists will be necessary.

### Follow-up

The patient has been receiving routine follow-up visits every three months in the orthopedic, urology, pediatric, and physiotherapy departments for the past year. The patient is now 2 years old, capable of sitting up without support, and can move around by crawling. There is no tone in the bilateral lower limbs, with diminished power of ⅖ and ⅕ in the left and right lower limbs, respectively. Fecal and urinary incontinence are still present while crying.

## Discussion

Although the exact cause of CRS is still unknown, there is a well-established link to maternal diabetes^1,7^. Chromosomal abnormalities, hereditary disorders, vascular hypoperfusion, and exposure to minoxidil and trimethoprim-sulfamethoxazole are other potential contributors^8^. A probable link between organic fat solvent exposure during pregnancy and sacral agenesis has been observed^9^. Familial instances point to a genetic etiology, and Currarino syndrome, a dominant hereditary sacral agenesis, is associated with the disease-causing HLXB9 gene, located at 7q36^10,11^. In our case, the mother was nondiabetic, with no history of diabetes in her family and no history of teratogenic drug consumption during pregnancy.

Defects in secondary neurulation before the fourth week of fetal life are believed to be the cause of sacral agenesis^12^. The caudal cell mass, which originates from the primitive streak and is responsible for the development of the spinal cord and vertebral bodies in the lower sacral region, is also involved in the formation of surrounding structures such as the genitourinary tract and anorectal organs^13,14^. As a result, congenital abnormalities including rectal stenosis, imperforate anus, epispadia, hypospadia, bifid scrotum, renal agenesis, horseshoe kidney, or low-lying kidneys may be associated. It is also linked to a defect in the abdominal wall closure, which results in gastroschisis and omphalocele. The vertebral abnormality can vary from simple coccygeal agenesis, with minimal neurological deficits, to total lumbosacral agenesis, with neurological compromise of the lower limbs, bowel, and bladder^15^. Because of its multisystemic involvement, sacral agenesis is a component of multiple syndromes, including VACTERL (vertebral defects, anal atresia, cardiac defects, tracheo-esophageal fistula, renal anomalies, and limb abnormalities), OEIS (omphalocele, cloacal exstrophy, imperforate anus, spinal abnormalities), and the Currarino triad (caudal agenesis, presacral mass, anorectal anomalies)^14^. In this case, kyphosis, lower limb flexion contractures, and clubfoot (CTEV) are the orthopedic manifestations. She also had urinary and fecal incontinence.

The majority of cases are discovered during a prenatal checkup or at delivery^16^. During the first trimester ultrasonographic scan, cases of sacral agenesis are discovered to have a shorter crown rump length than expected, a protuberance of the lower spine, and a large nuchal translucency^8,17^. The first trimester diagnosis is challenging due to inadequate sacral ossification^8^. Hence, the diagnosis is commonly made in the later stages of pregnancy by demonstrating the termination of the lumbar spine and abnormal lower extremities in the ʻVʼ attitude, ‘Buddha’s attitude’ or the ʻfrog-likeʼ position^15,16,18,19^. MRI is particularly useful in the context of oligohydramnios and maternal obesity, and it may be used to examine genitourinary, gastrointestinal, and musculoskeletal defects that may be associated^8^. Sacral agenesis has been detected as early as 11 weeks of pregnancy^19^. However, no defects were identified during prenatal ultrasound in this case.

The primary goal of treatment is to make the patient’s life easier. Management is challenging and necessitates a multidisciplinary approach, most commonly requiring urologic and orthopedic assistance. It is commonly associated with neurogenic bladder dysfunction, requiring urodynamic assessment at diagnosis and long-term urodynamic management^20^. Various orthopedic approaches have been described to manage spinopelvic instability. Griffet *et al*.^21^ reported lumbopelvic distraction and stabilization with external fixation in a boy, which allowed the boy to sit and walk with crutches. In another study, three patients who underwent a technique of posterior lumbopelvic instrumentation and fusion yielded an aligned spine with a posture that allowed sitting on the ischial spines^22^. Ferland *et al*.^23^ reported a 2-stage surgery of spinopelvic fusion augmented with a vascularized rib graft spanning the lumbo-pelvic junction in six patients, achieving a solid spinal and spinopelvic fusion in all six patients. Since our patient is just 1-year-old, she is yet to establish bladder and bowel control. If her symptoms persist, she should undergo intermittent urethral catheterization, or mitrofanoff for urinary symptoms, and rectal enemas, or malone for gastrointestinal symptoms^24^.

The degree of vertebral anomalies and the related abnormalities determine the prognosis. It is not associated with cognitive impairment, and people with less severe disorders that do not affect other systems can live a normal life; nevertheless, abnormalities in the bladder, colon, and lower limbs cause serious morbidity^15^.

Long-term follow-up is required for orthopedic and urologic assessments for respective functional impairments and improvements. The patient’s growth and developmental milestones are also checked at every follow-up visit.

## Conclusion

The current case emphasizes the rarity of CRS in a fetus of a nondiabetic mother. As there is no intrauterine intervention and postnatal therapy is primarily supportive, prevention becomes the sole option. Genetic and prepregnancy counseling in high-risk categories, such as diabetic mothers, offers hope. Early diagnosis and imaging are essential, with prenatal ultrasound screening being a valuable tool for detecting sacral agenesis.

### Limitation

No genetic analysis was performed as there is no facility for proper gene analysis in our hospital.

## Ethical approval

Ethical approval for this study is not required by the Kathmandu University School of Medical Sciences-Institutional Review Committee (KUSMS-IRC).

## Consent

Written informed consent was obtained from the patient’s mother for the publication of this case report and accompanying images. A copy of the written consent is available for review by the Editor-in-Chief of this journal on request.

## Sources of funding

Funding was not received for this study.

## Author contribution

J.T.: concept, data collection, manuscript preparation, edit and review, and guarantor; A.P.data collection as well: manuscript preparation, edit, and review; A.P.data collection as well: manuscript preparation, obtaining consent from the patient, edit and review; S.K., K.B., and A.C.: manuscript preparation, edit, and review

.

## Conflicts of interest disclosure

All authors declare that they have no conflicts of interest.

## Research registration unique identifying number (UIN)

1. Name of the registry: not applicable.
2. Unique identifying number or registration ID: not applicable.
3. Hyperlink to your specific registration (must be publicly accessible and will be checked): not applicable.

## Guarantor

Dr Jagadish Thapa MS Orthopedics, Spine fellow, Department of Orthopedics, Dhulikhel Hospital Dhulikhel, Kavre, Nepal.

## Data availability statement

Data sharing is not applicable to this article.

## Provenance and peer review

Not invited.
